# Inactivation of the *CMAH* gene and deficiency of Neu5Gc play a role in human brain evolution

**DOI:** 10.1186/s41232-025-00368-3

**Published:** 2025-02-08

**Authors:** Yuxin Liu, Jinhong Li, Qicai Liu

**Affiliations:** 1 Center of Reproductive Medicine, The First Affiliated Hospital, Fujian Medical University, Fuzhou, P.R. China; 2 Department of Laboratory Medicine, Medical Technology and Engineering College, Fujian Medical University, Fuzhou, P.R. China; 3 Vanke School of Public Health, National Graduate College for Engineers, Tsinghua University, Beijing, P.R. China; 4 Key Laboratory of Clinical Laboratory Technology for Precision Medicine (Fujian Medical University), Fujian Medical University, Fuzhou, P.R. China; 5 School of Biomedical Engineering, Tsinghua University, Beijing, P.R. China; 6 Department of Reproductive Medicine Centre, The First Affiliated Hospital, Fujian Medical University, 20 Chazhong Road, Fuzhou, 350005 China

**Keywords:** Neu5Gc, Brain evolution, Immunosenescence

## Abstract

During human evolution, some genes were lost or silenced from the genome of hominins. These missing genes might be the key to the evolution of humans’ unique cognitive skills. An inactivation mutation in CMP-*N*-acetylneuraminic acid hydroxylase (*CMAH*) was the result of natural selection. The inactivation of *CMAH* protected our ancestors from some pathogens and reduced the level of *N*-glycolylneuraminic acid (Neu5Gc) in brain tissue. Interestingly, the low level of Neu5Gc promoted the development of brain tissue, which may have played a role in human evolution. As a xenoantigen, Neu5Gc may have been involved in brain evolution by affecting neural conduction, neuronal development, and aging.

**Graphical Abstract:**

During human evolution, humans lost the ability to synthesize Neu5Gc after the inactivation mutation of the gene *CMAH*. Therefore, Neu5Gc in the human body is a xenoantigen. The inactivation of *CMAH* and the loss of endogenous Neu5Gc may have played a role in human brain evolution by affecting neural conduction, neuronal development, and aging.

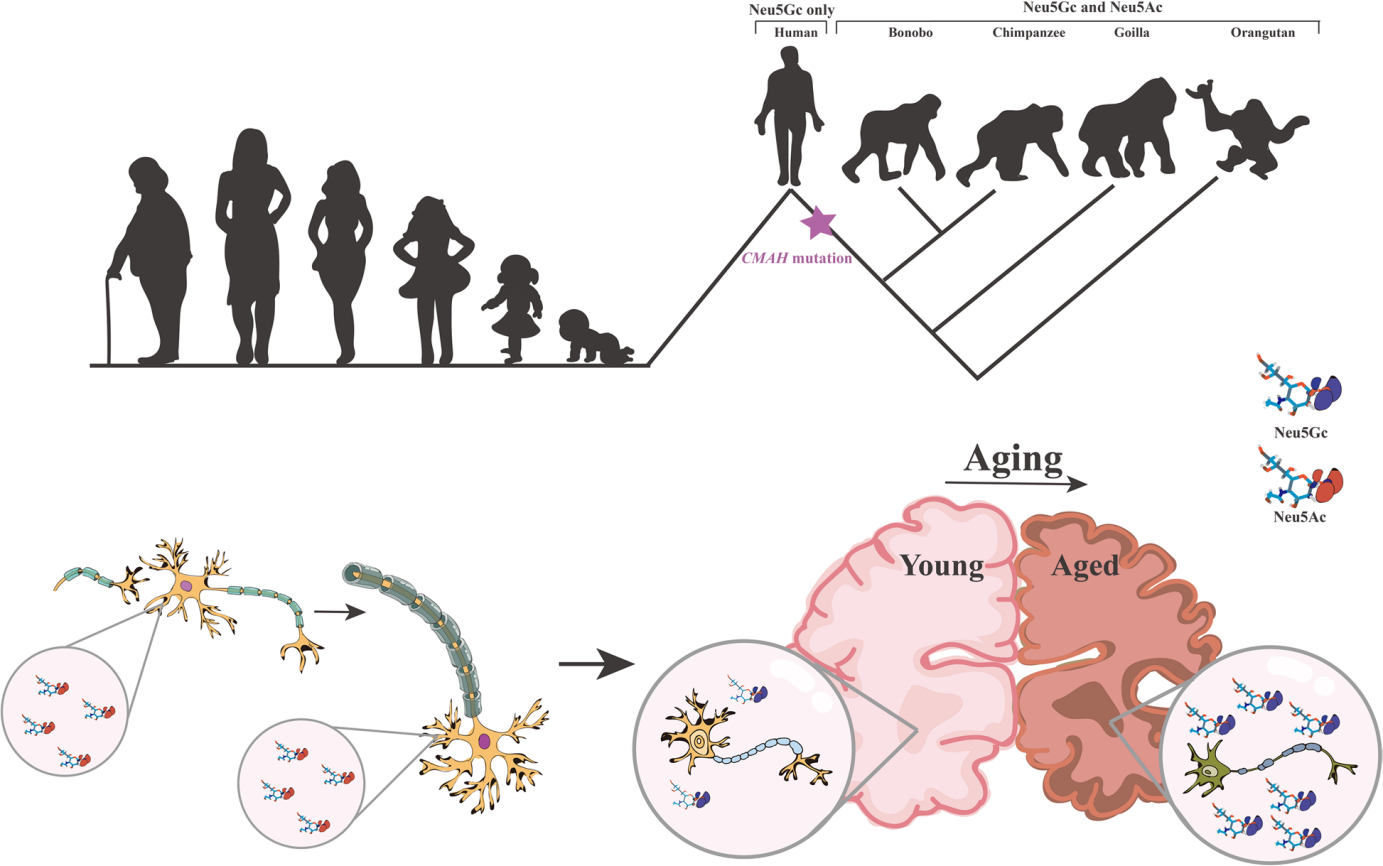

## Key points

• What is already known about this topic?

About 2.8 million years ago, the gene *CMAH* was inactivated in humans. Since then, Neu5Gc can no longer be synthesized in humans and is therefore a xenoantigen. Neu5Gc from dietary sources such as red meat and dairy products can be incorporated into glycoconjugates of cells. Therefore, low levels of Neu5Gc are present in the human body and continuously stimulate the immune system, including in the brain.

• What does this study add?

This review describes the adverse effects of Neu5Gc on brain development. The absence of endogenous Neu5Gc may play an important role in the evolution of the human brain. Abnormal accumulation of Neu5Gc in the brain is associated with aging and abnormal development of the brain. Neu5Gc-associated sialic acid (Sia) dysfunction can lead to abnormal brain connectivity. Moreover, high level of Neu5Gc is related to abnormal axonal myelination, Alzheimer’s disease (AD), and memory loss.

## Introduction

During human evolution, some genes have been mutated, deleted, or silenced due to environmental or reproductive selection, while most genes have been preserved and stabilized because they are adaptive [1]. These ancient genes have important physiological and even pathological implications for humans today. For example, some genes may be associated with genetic diseases in modern humans, such as depression and nicotine addiction [2, 3]. At the same time, some genes were silenced and disappeared from the human genome, which influenced metabolism, the immune system and nervous system, and even intelligence and aging in humans [4]. In early 2022, researchers made a major breakthrough in xenogeneic heart transplantation by developing “Pig 3.0,” whose glycan epitopes that are incompatible with the human immune system (galactose-*α*−1,3-galactose, *N*-glycolylneuraminic acid (Neu5Gc), and SDa epitopes) were knocked out. “Pig 3.0” is suitable for cross-species transplantation [5]. There was an inactivation mutation in CMP-*N*-acetylneuraminic acid hydroxylase (*CMAH*) in humans. The inactivation of CMAH has been shown to affect some systems in the human body, such as the cardiovascular system. This is closely related to a series of immune reactions triggered by Neu5Gc in human body [6–8]. The level of Neu5Gc in brain tissue was reduced after the mutation of *CMAH* [9]. Interestingly, the inactivation of *CMAH* occurred about 2.8 million years ago, and the expansion of the human brain volume occurred about 2.1–2.2 million years ago [10]. The timing of these evolutionary events suggests that Neu5Gc may have played a role in human brain evolution.

## Mystery of the loss of endogenous Neu5Gc

### Neu5Ac and Neu5Gc in nature

All vertebrate cells in nature are covered in a dense and complex layer of sugar chains whose ends are modified by abundant sialic acids (Sias) [11]. Sia is a hydroxylated monosaccharide acylation derivative with a backbone of nine carbon atoms that plays an important role in mediating cell recognition and cell flow, making it a bridge between cells and the extracellular matrix [12, 13]. At present, Sia has been found to be composed of *N*-acetylneuraminic acid (Neu5Ac), Neu5Gc, deaminoneuraminic acid, and their derivatives. These Sias are modified by methylation, acetylation, and sulfation at sites 4, 7, 8, and 9 to produce more than 50 chemical species [14]. Neu5Ac and Neu5Gc are the most common Sias on mammalian cells. Neu5Gc is produced when CMAH catalyzes a hydroxyl addition of Neu5Ac [15].

### Human evolution and inactivation of CMAH

A comparison of the genomes of Neanderthals, modern humans, and apes has revealed that some of the functional effects of DNA fragments unique to modern humans helped us evolve unique cognitive skills, such as a mutation in the *FOXP2* transcription factor 200,000 years ago, which had an important influence on motor control and language production in modern humans [16]. An inactivation mutation in the *CMAH* gene 2.8 million years ago may have contributed to human evolution [10]. The mutation consisted of a 92-bp deletion in the *CMAH* sequence, resulting in premature stop codon and functional inactivation of the enzyme [17]. Since then, humans can synthesize Neu5Ac but cannot modify it into Neu5Gc, whereas gorillas and ancient hominids can [18].

### Pathogen pressure and loss of endogenous Neu5Gc

Recent studies have shown that humans are not the only species to have lost the ability to synthesize Neu5Gc. Other mammals, such as some primates, bats, and toothed whales, also experienced the loss of function of the *CMAH* gene caused by exon deletion, a premature stop codon, or frameshift mutation [19–21]. This evidence suggests that the loss of endogenous Neu5Gc and the production of anti-Neu5Gc antibodies might be the result of natural selection, and that such genetic changes are adaptive. One possible explanation is that these mutations confer resistance to pathogens [22]. Pathogenic bacteria, protozoa, viruses, and toxins bind to host Sia to mediate invasion of cells [23, 24]. Therefore, human ancestors escaped infection by nonhuman hominid (NHH) malaria, a pathogen with a preference for binding to Neu5Ac and its derivatives, by eliminating the synthesis of Neu5Gc [25]. Although a strain of this NHH malaria later evolved to preferentially bind to human Neu5Ac-rich red blood cells, now known as human *Plasmodium falciparum* malaria [26], the evolution of NHH malaria also explained the difference in Sia-binding preference between human *P. falciparum* malaria parasites and African NHH erythrocytes [17].

### Reproductive compatibility and loss of endogenous Neu5Gc

**Fig. 1:**
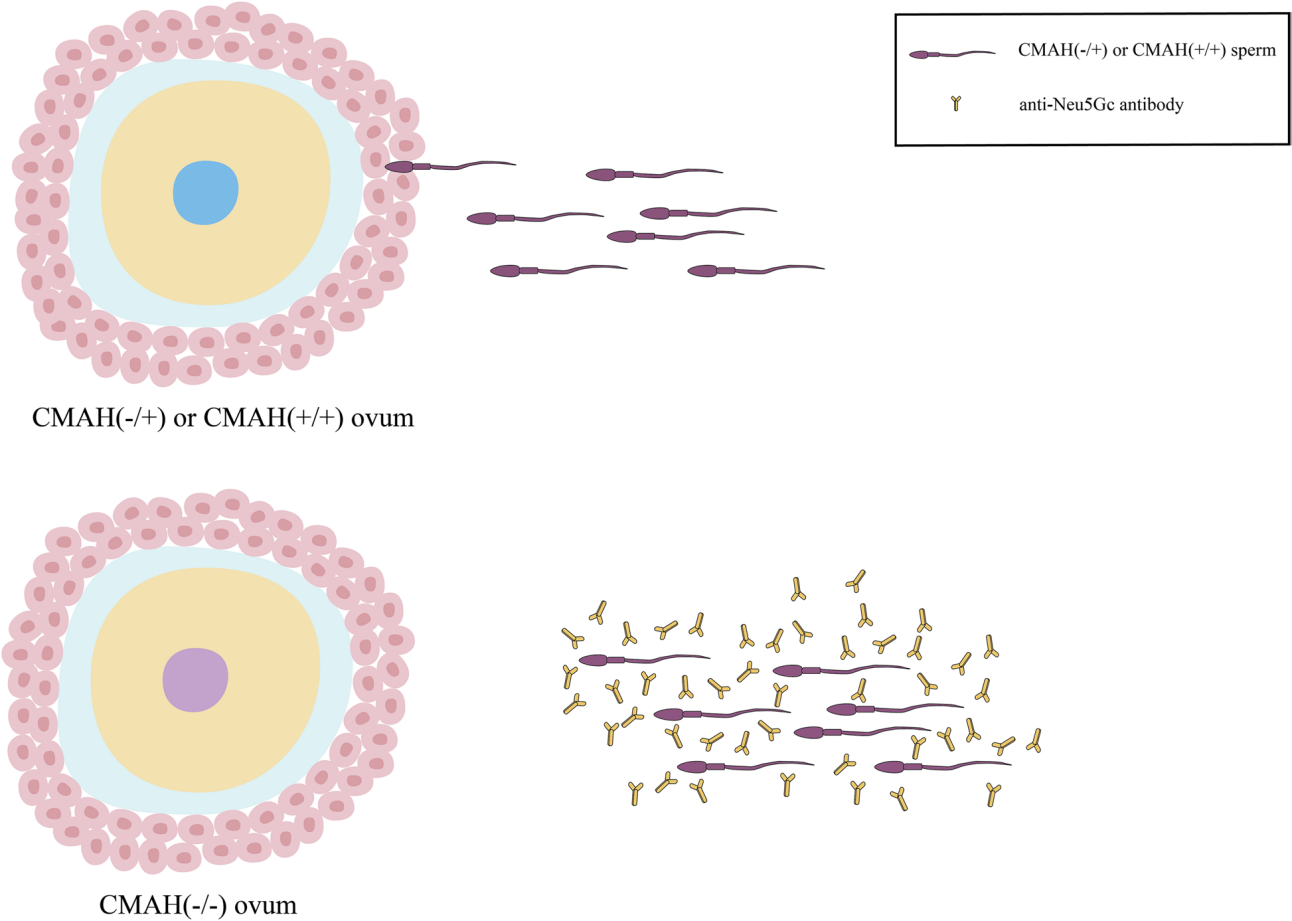
Schematic diagram of the reproductive selection by human Sia. *CMAH* (−/−) females develop antigenic immunity to Neu5Gc-expressing sperm produced by *CMAH* (+/−) or *CMAH* (+/+) males due to the presence of anti-Neu5Gc antibodies. In addition, *CMAH* (−/−) females favor the sperm produced by *CMAH* (−/−) males. This reproductive conflict will be conducive to the constant rise and fixation of *CMAH* (−) allele frequencies in the population

Another intriguing explanation for the inactivation of *CMAH* is that selective reproduction between people who lack endogenous Neu5Gc may lead to positive selection for this genotype [27]. In the sperm of *CMAH* (− / +) or *CMAH* (+ / +) male mice with normal *CMAH* gene function, Neu5Gc will be carried by a highly sialic GPI-anchored protein such as CD52 [28]. However, *CMAH* (− / −) female mice with inactivation of *CMAH* gene function can produce anti-Neu5Gc antibodies in the reproductive tract that bind to sperm carrying Neu5Gc. This immune reaction will lead to the majority of sperm being destroyed by uterine immune cells, thus seriously reducing fertility (Fig. 1) [26, 27]. This reproductive xenoimmunity can drive the frequency of the *CMAH* ( −) allele up in the population to fixation.

### Brain evolution and loss of endogenous Neu5Gc

According to the current scientific consensus, the evolution of ancient hominids into modern humans and modern NHHs involved complex gene-environment interactions at the population level. The inactivation of the *CMAH* gene, driven by a combination of pathogen avoidance and reproductive conflict, would theoretically contribute to human survival and development. Although this mutation would have greatly changed the molecular composition of glycosylation in cells throughout the human body, it conferred the unique cognitive and physical adaptations of modern humans compared to NHHs [29]. Some studies have found that Neu5Gc might have a toxic effect on the vertebrate nervous system, thus affecting the evolution of the brain [30]. However, endogenous Neu5Ac is expressed in the human nerve cell membrane at a level 2–4 times higher than in most other mammals [31]. This could mean that the complete loss of endogenous Neu5Gc in brains rich in Sia might have helped humans evolve more complex and plastic brains. In addition, humans have the ability to run long distances, which is unique among primates. This ability contributed to an increased range of resource exploration, the pursuit of prey over long distances, and escape from danger [32]. We attribute this human high endurance to anatomical and physiological adaptations [33, 34]. Some studies have found that the inactivation of the *CMAH* gene in mouse models can promote the ability to use oxygen and fatigue resistance in muscle, which lead to increased endurance [35].

## Absorption and metabolism of Neu5Gc

**Fig. 2:**
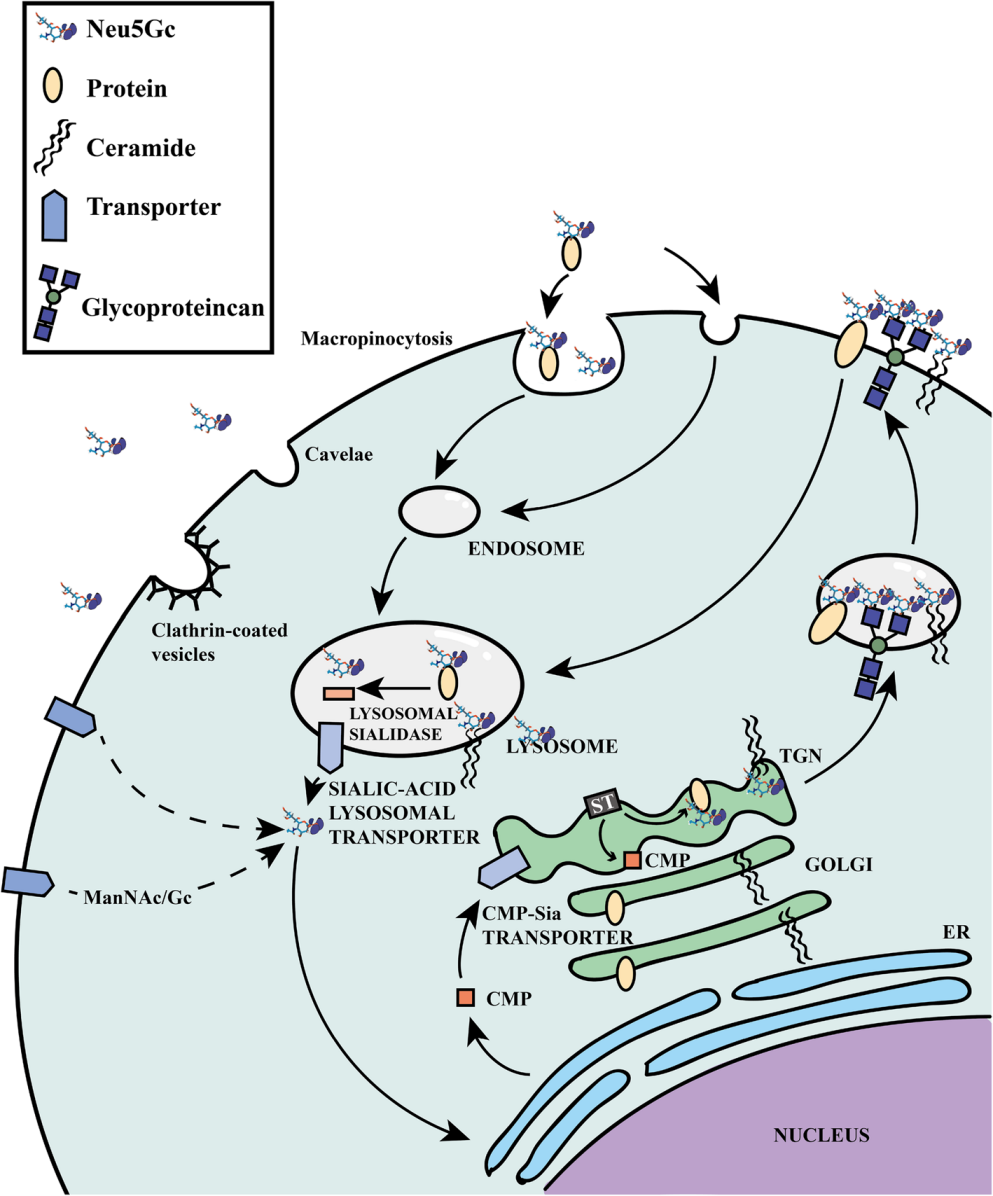
Pathway of uptake and expression of Neu5Gc in human cells. Neu5Gc is expressed on the surface of human cells mainly through the processing and integration of lysosome, nuclear, and Golgi bodies

Due to the inactivation of the *CMAH* gene, Neu5Gc cannot be synthesized in humans. However, trace amounts of Neu5Gc can still be detected in human cells [36]. *CMAH* (− / −) mice exhibit a complete absence of Neu5Gc throughout the body, suggesting a lack of alternative pathways for Neu5Gc synthesis in the human body [37, 38]. An alternative explanation is that exogenous Neu5Gc can be introduced into human tissues. Dietary sources rich in Neu5Gc mainly include red meat and dairy products [39]. In fact, the human metabolic system does not discriminate between Neu5Gc and Neu5Ac. It recognizes exogenous Neu5Gc as Neu5Ac [36, 40, 41]. Because of this, exogenous Neu5Gc can be incorporated into epithelial cells, endothelial cells, embryonic cells, and cancer cells [40, 42–44]. First, free Neu5Gc is absorbed by endocytosis in the gut, and bound Neu5Gc is released by lysosomal sialidase. Then Neu5Gc is transported to the cytoplasm by Sia transporters and is activated as CMP-Neu5Gc in the nucleus. Finally, CMP-Neu5Gc enters the Golgi body through Sia transporters where sialyltransferase can transfer Neu5Gc to newly synthesized glycoconjugates. Finally, Neu5Gc is expressed on the cell surface [41]. Exogenous Neu5Gc that is absorbed into the body is incorporated into glycosides, while the free form of Neu5Gc is utilized by intestinal microorganisms or rapidly cleared by the kidneys through urine [45]. Furthermore, the Neu5Gc level in the human body usually remains low [36], so physiological mechanisms must prevent excessive accumulation. Enzyme mechanisms have been found in human cells that convert Neu5Gc to *N*-glycolyl mannosamine, *N*-glycolyl glucosamine, and finally *N*-glycolyl glucosamine 6-phosphate. Irreversible de-*N*-glycosylation of *N*-glycolyl glucosamine 6-phosphate forms the ubiquitous metabolite glucosamine 6-phosphate (GlcNH_2_−6-P), which can enter glycolysis through further conversion to fructose 6-phosphate, glucose 6-phosphate, and glycolic acid. These metabolites can enter the citric acid cycle through glyoxylic acid, ultimately maintaining exogenous Neu5Gc at a healthy level (Fig. 2) [45]. The study also clarified the heterogeneous expression of Neu5Gc in tissues and cells, which will provide clues for the study of specific target antigens for tumors or autoimmune diseases [36].

## The immune damage of Neu5Gc to the human brain

### Neu5Gc aggravates the risk of immune damage to the blood–brain barrier

Unlike other heteroglycans, Neu5Gc is characterized by its dietary source, antigenicity of the monomer itself, and human cell surface abundance, so its deficiency may cause extensive immunological effects in the human body [46]. Although human metabolism cannot discriminate between Neu5Ac and Neu5Gc, the human immune system can. Humans in a clinical trial contained different levels and types of circulating Neu5Gc-specific immunoglobulin [47, 48]. Human anti-Neu5Gc antibodies interact with Neu5Gc to promote chronic inflammation and ultimately lead to the occurrence and development of various human diseases [49]. The ability of exogenous Sia to cross the blood–brain barrier (BBB) and incorporate into Sia-sugar conjugates in different brain regions has been observed by oral and intravenous administration of radiolabeled Sia in rodents and newborn pigs [50, 51]. It can be inferred from isotopic studies that dietary-derived Sia first enters the blood and crosses the BBB by diffusion or by protein receptor-mediated processes. In the brain, dietary Neu5Gc can be incorporated into important regions of the central nervous system (CNS), such as the BBB and axon-myelin units, creating targets for anti-Neu5Gc antibodies and resulting in changes in BBB permeability and instability of axon-myelin coupling [52].

### Immune damage to the blood–brain barrier leads to neuroinflammation

Under normal circumstances, the brain is separated from the rest of the body by the BBB and therefore has immune privilege. However, in some pathological cases, the BBB integrity will be damaged, which manifests as increased permeability, thus enabling communication between the peripheral and central immune system [53]. Inflammation is a major factor affecting the structure and function of the BBB [54]. Studies have shown that biological mediators secreted into the blood during peripheral chronic inflammation may damage the BBB, triggering CNS diseases [55]. A damaged BBB leads to entry of other systemic myeloid cells into the CNS, thereby enhancing brain inflammation [56]. Increased neurodegeneration has been observed in an animal model with persistent inflammatory neurodegeneration after peripheral inflammatory stimulation [57].

Another possible way in which the peripheral and central immune systems interact is via neurotransmission. Rodent models have indicated that the vagus nerve is capable of transmitting information about the inflammatory state of the body to the brain and increases the levels of brain cytokines in the case of persistent peripheral inflammation [58]. Proinflammatory cytokines lead to the activation of neurogliocyte and perivascular macrophages, initiating or contributing to neuroinflammation. Enhanced neuroinflammation promotes the development of several highly prevalent neurological diseases, mainly Alzheimer’s disease (AD) [59], Parkinson’s disease [60], and multiple sclerosis (MS) [53].

### Neu5Gc damaged the normal physiological function of Neu5Ac

#### The role of Neu5Ac in brain development

Studies have shown that Neu5Ac plays an important role in brain development. Neu5Ac is involved in the formation of ganglioside GM1 and polysialic acid (polySia, PSA), which take part in neuronal differentiation, growth, and regeneration and support synaptic transmission, thus affecting learning and cognitive ability [61]. In the process of learning and cognition, neurons need to exchange a great deal of information quickly in the brain. This cannot be achieved without the synaptic interaction between neurons [62]. The formation of new synapses is a hallmark of learning. Sias in human body, mainly Neu5Ac, are essential for synapse formation. In a mouse model, exogenous supplementation of Neu5Ac during pregnancy increased the level of Neu5Ac in the cerebral cortex and hippocampus, brain areas involved in learning and memory, of offspring mice. The offspring mice performed better in learning and cognitive experiments [61, 63, 64]. In addition, if the gene N-acetylneuraminate synthase (*NANS*) encoding Neu5Ac synthase is mutated, severe developmental delay occurs in infants [65], suggesting that the endogenous synthesis of Neu5Ac is critical for brain development.

However, if exogenous Neu5Ac is directly given to offspring mice after birth, the learning and cognitive ability of offspring mice does not improve [61]. This may be because brain development is time-dependent. Once the brain’s growth has passed its peak, it cannot be rebooted, which can have a big impact on cognitive function in adulthood [66]. And the synthesis and transport of Neu5Ac play a role mainly in the early ontogenetic stage of rapid brain development.

#### Incorporation of Neu5Gc disrupts the normal physiological function of Neu5Ac

Observational and clinical studies have shown that a diet rich in red meat increases the risk of neurodegenerative diseases [67]. In this part, we will try to explain from the perspective that the incorporation of Neu5Gc in the diet will destroy the normal physiological function of Neu5Ac. Neural cell adhesion molecule (NCAM) is an important molecule for Neu5Ac to function in the brain. PSA-NCAM acts as a modulator of brain plasticity, promoting repair and regeneration after neurological damage [68]. PSA deficiency causes severe neural phenotypes in mice, such as defective neuronal network connectivity, abnormal localization of neurons and glial cells, and glial cell differentiation, which can be alleviated by depletion of NCAM [69]. These severe phenotypes suggest that Neu5Ac is closely associated with NCAM and plays an important role in normal physiological function. In addition, PSA can combine neurotrophins such as brain-derived neurotrophic factor (BDNF), nerve growth factor, neurotrophin-3, and neurotrophin-4. Combined with polySia, BDNF can bind to and activate its receptors [70]. However, the presence of Neu5Gc has been reported to affect the degradation of PSA by an endogenous sialidase, Neu1. And Neu1-induced BDNF-related release is inhibited [71].

#### Anti-Neu5Gc antibody affects the normal physiological function of Neu5Ac

**Fig. 3:**
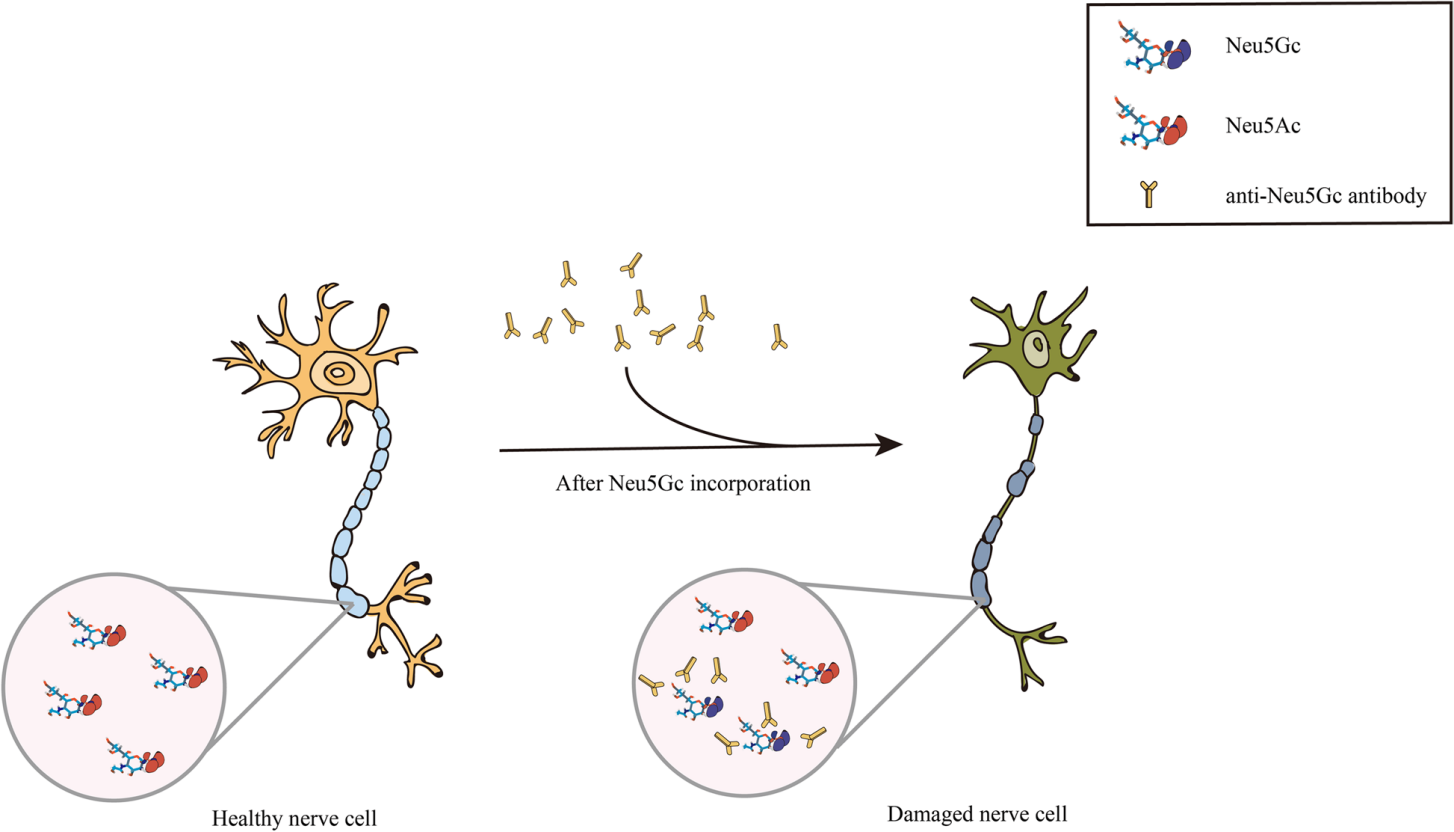
Schematic diagram of cross-reaction between anti-Neu5Gc antibody and Neu5Ac. As an important component of GM1, Neu5Ac is highly similar to Neu5Gc in structure. There may be cross-reaction between anti-Neu5Gc antibodies and Neu5Ac, resulting in nerve cell damage

Dietary Neu5Gc and circulating anti-Neu5Gc antibodies may interact in central and peripheral nervous system and influence the occurrence and development of CNS diseases, suggesting that a diet high in Neu5Gc may be an overlooked environmental risk factor for CNS diseases [72]. MS is the most common demyelinating disease of the CNS. Its pathogenesis is caused by multiple factors. Interestingly, this disease seems to be found only in humans, and not in chimpanzees [73]. Therefore, It has proposed that dietary Neu5Gc is incorporated into the nervous system, and because it is a xenoantigen, the immune system produces anti-Neu5Gc antibodies against it [73]. These circulating antibodies may cause damage to the BBB, myelin sheath, axon, and other structures in the CNS, making the CNS environment unstable, thus raising the risk of MS [73]. Current study suggested that the CNS damage in MS is mainly caused by immune factors. Primary infection of infectious mononucleosis (IMN) may impair the integrity of BBB, during which anti-Neu5GC antibodies increase. This phenomenon is also consistent with the viewpoint of BBB damage mentioned above [74]. What’s more, Boligan et al. have found that IgG deposition can be observed in the lesion sites of MS, and that IgG antibodies in the serum and CSF of MS patients show increased reactivity to Neu5Gc and Neu5Ac [47]. Notably, a high degree of overlap in IgG reactivity to Neu5Gc and Neu5Ac in individual patients was found in this study. Neu5Gc and Neu5Ac are highly similar in structure, only differing by one oxygen atom, so it is reasonable to suspect that the increased IgG reaction may be caused by cross-immune reaction (Fig. 3) [75].

## Neu5Gc and brain function

### Abnormal sialylation and impaired brain function after loss of Neu5Gc

Brain gangliosides and Neu5Ac play crucial roles in cell–cell interactions, neuronal growth, modification of synaptic connectivity, and memory formation [76]. However, the accumulation of Neu5Gc, which is only one oxygen atom apart, in the brain causes abnormal sialylation. Mice with abnormal cerebral sialylation developed myelin deformity, with 40% reduction of major myelin proteins, 30% reduction of myelinated axons, 33% reduction of myelin thickness, and disruption of nodes of Ranvier molecules. What’s more, these mice exhibited impaired motor coordination, gait disturbances, and severe cognitive impairment [77]. In addition, Since Neu5Gc cannot be synthesized in humans and can induce an immune response, its presence is important for controlling sialylation of complex glycoproteins [78]. In a study, the Sia state of macrophages was regulated by feeding exogenous-free Sia (Neu5Ac, Neu5Gc) and sialidase inhibitors to cells, and their effects on cell mechanics and function were detected [79]. Over-activated microglial phagocytosis of neurons and synapses could lead to neurodegenerative diseases, while high sialylation of the neuron cell surface inhibits microglial phagocytosis of such neurons [80]. Neu5Gc-modified transferrin exacerbates iron loading-associated amyloid-β cytotoxicity which is rescued by Neu5Ac-modified transferrin [72].

### Loss of endogenous Neu5Gc and connectivity in the brain

A recent issue of the journal *Science* has suggested that the key to brain function is the communication between different brain regions, in other words, brain connectivity [81]. In the human cortex, excitation-excitatory synaptic dynamics differ from those in the mouse cortex and vary with the depth of second and third layers [82]. Without smoothly functioning connections, brain function will be greatly affected. The early stages of infant neurodevelopment are critical for establishing neural structures and synaptic connections, and breast milk is rich in Neu5Ac, which is compatible with the needs of rapid brain development in infancy [61, 63, 64, 83]. Diets rich in Sia can increase the level of Sia in the brain of newborn piglets, increase the expression level of learning-related genes, and enhance learning and memory ability [83]. Studies involving synthetic Neu5Ac and Neu5Gc polymers have shown that mammalian and bacterial sialidase have a much lower ability to hydrolyze α2–8-linked Neu5Gc at the nonreducing end. The resistance of Neu5Gc-containing polySia to sialidase provides a possible explanation for the low level of Neu5Gc in vertebrate brains [84]. In a study, single oxygen atom changes were introduced into polySia by an exogenous non-neurogenic Sia, Neu5Gc, which induced resistance to polySia turnover induced by sialidase 1 and inhibited the release of brain-derived neurotrophins associated with it [71]. Neu5Gc on the surface of the macrophage can regulate phagocytosis and secretion of inflammatory factors by resisting sialidase 1-mediated polySia degradation [85]. The binding of Neu5Gc leads to increased resistance to sialidase and abnormal function of Sia-rich cells, which might influence the communication speed between neurons by affecting the Sia structure on the surface of neurons, and influence behavior and cognitive ability by affecting brain connectivity.

#### Neu5Gc affects brain aging and development

Humans and chimpanzees share > 99% residue identity in most proteins [86]. However, a marked decline in cognitive flexibility has been observed in chimpanzees at an average age of 22.5 years that is not observed in humans [87]. A rare genetic difference between humans and chimpanzees is the human-specific inactivation of the *CMAH* gene, which modifies Neu5Ac to Neu5Gc. Abnormal accumulation of Neu5Gc is correlated with the aging and abnormal development of the brain [88]. At present, cognitive dysfunction induced by human brain aging is an important cause of decline in quality of life. Neu5Gc-related Sia dysfunction may lead to sialylation in brain tissue and abnormal brain connectivity [83], AD [72], and memory loss [88]. The evolutionarily conserved brain-specific inhibition of Neu5Gc synthesis may indicate that its presence is toxic to the organ [9], and that the inactivation mutation in *CMAH* may have played a role in human brain evolution [89]. To explore the consequences of forced expression of Neu5Gc in the brain, a brain-specific *CMAH* transgenic mice model was established. Overexpression of Neu5Gc in the brain led to abnormal motor activity, impaired object recognition memory, and abnormal myelination of axons [88]. Neu5Gc is present at significant levels in all dairy, including dairy-based infant formula, whereas only trace levels of Neu5Gc are present in human breast milk [40]. High brain ganglioside and glycoprotein Sia concentrations in infants fed with human milk suggest increased synaptogenesis and differences in neurodevelopment [90].

## Prospects

**Fig. 4:**
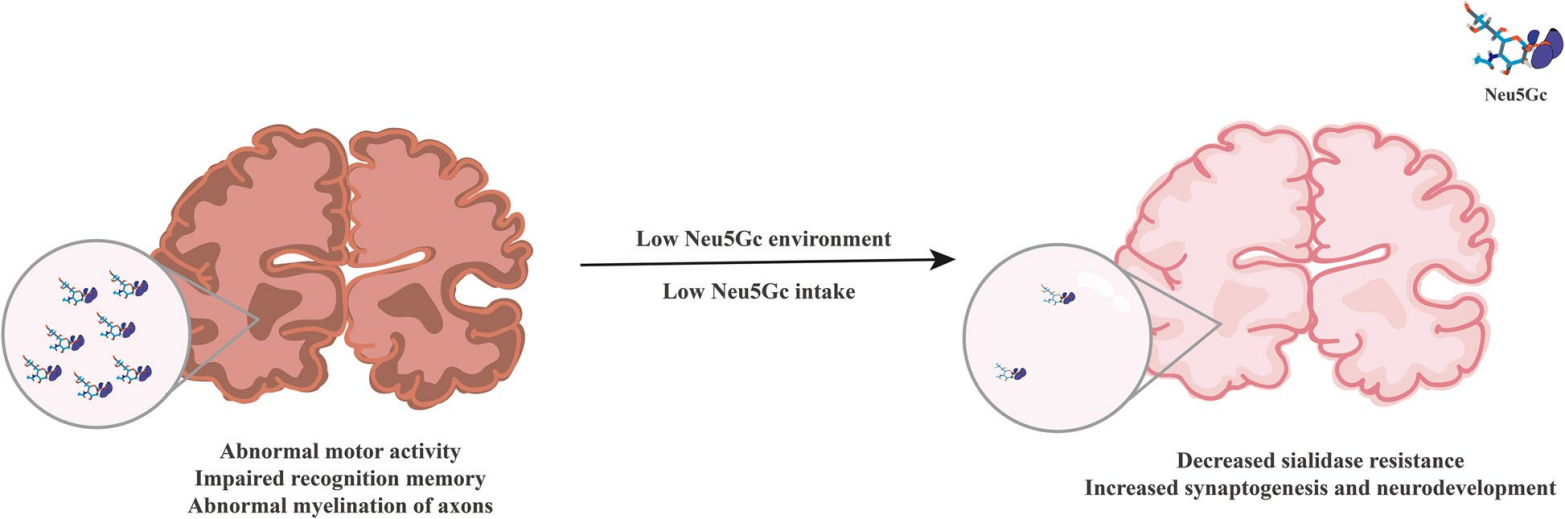
Prospects to create a lower Neu5Gc environment for the brain. The loss of Neu5Gc from the environment or reduced human intake may lead to improvements in motor performance, cognitive ability, and memory

First, Neu5Gc overexpression in the brain results in abnormal locomotor activity, impaired object recognition memory, and abnormal axon myelination [88]. Second, inhibition of Neu5Gc synthesis in the brains of most animals is an important prerequisite for normal brain function [88], and Neu5Gc levels are very low in the brains of all tested vertebrates [45]. What’s more, the timing of human brain evolution coincides with the time of the inhibition of Neu5Gc synthesis in humans [10]. Much circumstantial evidence has suggested that Neu5Gc inhibits brain function. At present, the literature shows that Neu5Gc in the human body mainly comes from diet, virus carriers, and biological products and mainly include red meat and dairy products. Ingested Neu5Gc is excreted by the kidneys, and only a small part is deposited in tissues such as the heart, liver, and muscle [85]. Current studies have found that endogenous Neu5Gc does not exist in the brain tissue of human or *CMAH* (− / −) mice, or that the concentration is very low. But some studies have shown that Neu5Gc has a strong ability to break through the BBB [91]. In view of the potential risk of Neu5Gc to brain development, as well as the characteristics of Neu5Gc intake and metabolism in the human population, a diet low in Neu5Gc-rich foods (such as red meat and dairy products) is a potential topic of research for preventing Neu5Gc accumulation in the human body and reducing the risk of brain aging in the future. Especially for neonates with rapid brain development, the control of Neu5Gc in dairy products may minimize the adverse effects of Neu5Gc on the brain and ensure a better start in early brain development (Fig. 4).

## Data Availability

This article has no additional data.
